# Predictive nomograms for early death in metastatic bladder cancer

**DOI:** 10.3389/fsurg.2022.1037203

**Published:** 2023-01-13

**Authors:** Tao Chen, Shuibo Shi, Ping Zheng, Xiangpeng Zhan, Ji Zhang, Yihe Li, Dongshui Li, Bin Fu, Luyao Chen

**Affiliations:** ^1^ Department of Urology, The First Affiliated Hospital of Nanchang University, Nanchang City, China; ^2^ Department of Urology, Shangrao Municipal Hospital, Shangrao City, China

**Keywords:** metastatic bladder cancer, SEER database, early death, prognosis, nomograms

## Abstract

**Background:**

Metastatic bladder cancer (MBC) is an incurable malignancy, which is prone to early death. We aimed to establish models to evaluating the risk of early death in patients with metastatic bladder cancer

**Methods:**

The data of 1,264 patients with MBC registered from 2010 to 2015 were obtained from the Surveillance, Epidemiology, and End Results (SEER) database. We utilized X-tile software to determine the optimal cut-off points of age and tumor size in diagnosis. Univariate and multivariate logistic regression analyses were used to identify significant independent risk factors for total early death and cancer-specific early death, then we construct two practical nomograms. In order to validate our prediction models, we performed calibration plots, receiver operating characteristics (ROCs) curves, decision curve analysis (DCA) and clinical impact curve (CIC).

**Result:**

A total of 1,216 patients with MBC were included in this study. 463 patients died prematurely (≤3 months), and among them 424 patients died of cancer-specific early death. The nomogram of total premature death was created by surgery, chemotherapy, tumor size, histological type, liver metastases, and nomogram of cancer-specific early death was based on surgery, race, tumor size, histological type, chemotherapy, and metastases (liver, brain). Through the verify of calibration plots, receiver operating characteristics (ROCs) curves, decision curve analysis (DCA) and clinical impact curve (CIC), we concluded that nomogram were a valid tool with excellent clinical utility to help clinicians predict premature death in MBC patients.

**Conclusions:**

The nomograms derived from the analysis of patients with MBC, which can provide refined prediction of premature death and furnish clinicians with useful ideas for patient-specific treatment options and follow-up scheduling.

## Introduction

Bladder cancer is a malignancy disease that occurs widely throughout the world. Most of all diagnosed patients with this disease is non-muscle invasive. 30% have an initial diagnosis of muscle-invasive bladder cancer and 5% a metastatic bladder carcinoma (1). Approximately 550,000 people suffer from this disease each year, nearly 200,000 people died from bladder tumors just in 2018 alone (2). MBC is often accompanied with a high cancer-specific-death rate, and the prognosis of MBC patients tends to be poor (3). In addition, patients with MBC are still at high risk of early death for a variety of reasons. Exploring these risk factors associated with premature death can help clinicians to predict premature death, develop individualized treatment plans, reduce the incidence of premature death and develop follow-up programmes.

To date, studies on premature death in patients with MBC has not been reported. Therefore, there is a great demand to develop a simple-to-use and practical tool to identify the risk factors leading to early death in MBC patients. Nomogram is a useful statistical model of integrate relevant factors to predict individual tumor prognosis (4), which have been widely used to assist physicians in developing treatment plans and assessing the prognosis of various cancers. The National Comprehensive Cancer Network guidelines have already introduced many nomograms with excellent performance (5).

The data for our study were obtained from the Surveillance, Epidemiology and End Results (SEER) database, the definitive cancer population registry in the US, which collects incidence and survival data for approximately 34.6% of all cancers in the US cancer registry. We obtained data of 1,264 patients with metastatic bladder cancer registered between 2010 and 2015 in the SEER, and used these to create two practical nomograms to assess its premature death, with the aim of helping clinicians to better develop their clinical work.

## Materials and methods

### Patients

**Figure 1:**
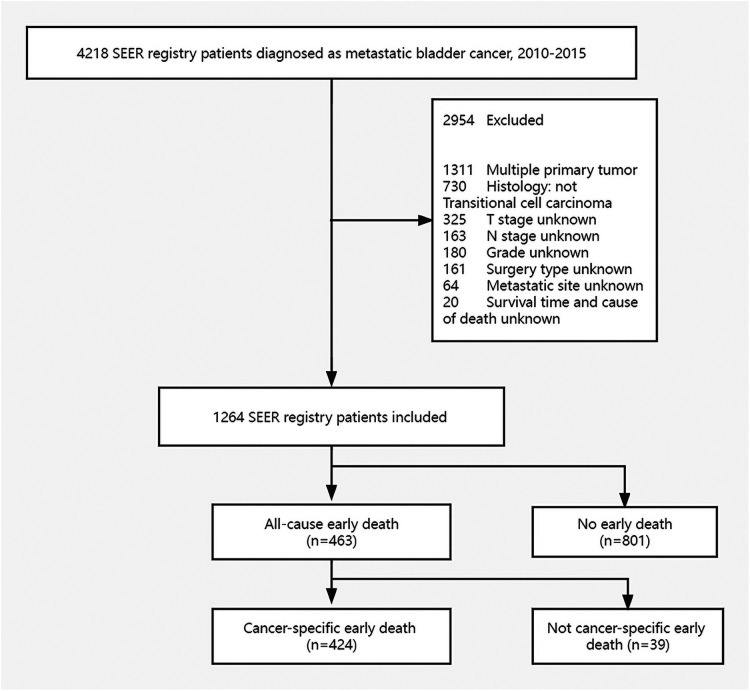
The flowchart of patients selection.

The data were extracted using SEER*Stat version 8.3.6.1. In the SEER database, patients with metastatic bladder cancer were registered from 2010 to 2015 according to the International Classification of Tumor Diseases Third Edition. The inclusion criteria were: (1) bladder cancer is the only one primary carcinoma and (2) Histology type is transitional cell carcinoma. Exclusion principles were: (1) unknown cause of death and survival time, (2) lack of metastatic site information, (3) unknown tumor size, (4) undetermined T and N stage and Grade, (5) unknown race and marital status, 6) lack of surgical information. The concrete screening process was shown in Figure 1.

Ultimately, we included the following variables: Age at diagnosis, gender, race, histological type, tumor size, grade, T-stage (AJCC 7th edition), N-stage (AJCC 7th edition), bone, lung, liver and brain metastases, surgery, radiotherapy, chemotherapy. Based on previous experience, we defined patients who dies within three months of diagnosis as early death (6, 7).

### Nomogram construction and statistical analysis

**Figure 2:**
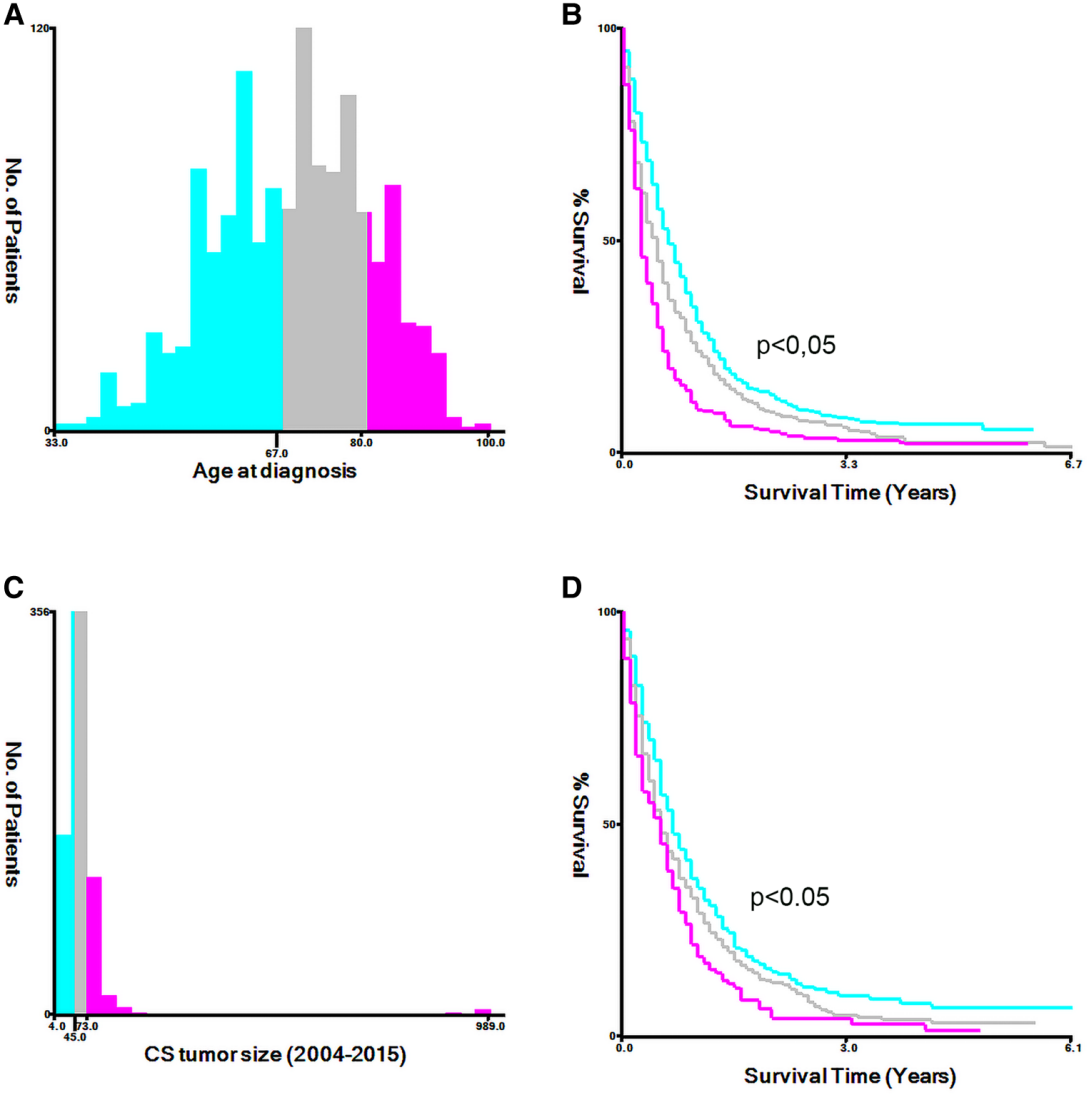
The appropriate cutoff values of age and tumor size was assessed by X-tile analysis. (**A,B**) The appropriate cutoff values of age were 67 and 80 years; (**C,D**) The appropriate cutoff values of tumor size were 45 and 73 mm.

For age and tumor size at diagnosis, we utilized the X-tile software to calculate the optimal cut-off points (Figure 2). The total cohort was randomly divided training and validation cohort in the ratio of 7 : 3, Univariate and multivariate logistic regression analyses were utilized to calculate the odds ratios (OR) and 95% confidence intervals (CI). Then, the identified risk factors were used to construct practical nomograms that can predict early death of MBC. Nomograms were evaluated by bootstrapping (1,000 resamples) to generate calibration plot (8) and the area under the curve (AUC) of the receiver operating characteristic curve (ROC) (9). The clinical benefit of nomograms was assessed by means of clinical decision curves (DCA) and clinical impact curve (CIC) (10). All statistical analyses were performed using SPSS (version 24.0; SPSS, Inc.), X-tile software, and software packages (rms, pROC, and rmda) in R software version 4.1.2. We measured two-tailed *p* values ≤ 0.05 to be statistically significant.

## Result

### Patient characteristics

A total of 1,264 patients with metastatic bladder cancer were included in the SEER database according to the inclusion and exclusion criteria. Among them, 463 people died prematurely, and 424 people died of tumor specificity. The majority of those who died prematurely were white (86.6%) and between the ages of 67 and 80 (41.3%). The most common pathological type was transitional cell carcinoma (74.5%). And the more common sites of tumor metastasis are bone (38.9%) and lung (40.0%). Most patients with early death received transurethral resection of bladder tumor (78.3%), without radiotherapy (80.7%) and chemotherapy (80.4%). The concrete characteristics of patients with MBC are shown in Table 1.

**Table 1:** Characteristics with metastatic bladder cancer patients.

Characteristic	SEER cohort (*n* = 1264)		
	No early death (%)	Total early death (%)	Cancer-specific early death (%)
All	801	463	424
**Age**			
<67	370 (46.2)	138 (29.8)	129 (30.4)
67–80	318 (39.7)	193 (41.7)	175 (41.3)
>80	113 (14.1)	132 (28.5)	120 (28.3)
**Sex**			
Male	597 (74.5)	320 (69.1)	296 (69.8)
Female	204 (25.5)	143 (30.9)	128 (30.2)
**Race**			
White	666 (83.1)	395 (85.3)	367 (86.6)
Black	91 (11.4)	52 (11.2)	42 (9.9)
Others	44 (5.5)	16 (3.5)	15 (3.5)
**Histologic type**			
PTCC	283 (35.3)	117 (25.3)	108 (25.5)
TCC	518 (64.7)	346 (74.7)	316 (74.5)
**Tumor size**			
<45 mm	166 (20.7)	58 (12.5)	51 (12.0)
45–73 mm	208 (26.0)	99 (21.4)	96 (22.6)
>73 mm	85 (10.6)	61 (13.2)	55 (13.0)
Unknown	342 (42.7)	245 (52.9)	222 (52.4)
**Grade**			
GI–II	21 (2.6)	15 (3.2)	14 (3.3)
GIII	189 (23.6)	134 (28.9)	119 (28.1)
GIV	591 (73.8)	314 (67.8)	291 (68.6)
**T stage**			
T1	136 (17.0)	102 (22.0)	93 (21.9)
T2	406 (50.7)	228 (49.2)	211 (49.8)
T3	99 (12.4)	42 (9.1)	38 (9.0)
T4	160 (20.0)	91 (19.7)	82 (19.3)
**N stage**			
N0	448 (55.9)	296 (63.9)	268 (63.2)
N1	101 (12.6)	49 (10.6)	45 (10.6)
N2	172 (21.5)	88 (19.0)	82 (19.3)
N3	80 (10.0)	30 (6.5)	29 (6.8)
**Bone metastasis**			
No	529 (66.0)	283 (61.1)	257 (60.6)
Yes	272 (34.0)	180 (38.9)	167 (39.4)
**Lung metastasis**			
No	548 (68.4)	278 (60.0)	253 (59.7)
Yes	253 (31.6)	185 (40.0)	171 (40.3)
**Liver metastasis**			
No	691 (86.3)	328 (70.8)	299 (70.5)
Yes	110 (13.7)	135 (29.2)	125 (29.5)
**Brain metastasis**			
No	781 (97.5)	445 (96.1)	407 (96.0)
Yes	20 (2.5)	18 (3.9)	17 (4.0)
**Surgery**			
No	53 (6.6)	83 (17.9)	77 (18.2)
TURBT	626 (78.2)	362 (78.2)	332 (78.3)
RC	122 (15.2)	18 (3.9)	15 (3.5)
**Radiotherapy**			
No	609 (76.0)	377 (81.4)	342 (80.7)
Yes	192 (24.0)	86 (19.0)	82 (19.30
**Chemotherapy**			
No	206 (25.7)	375 (81.0)	341 (80.4)
Yes	595 (74.3)	88 (19.0)	83 (19.3)

### Analysis of independent risk factors

Risk factors associated with early death of MBC patients were identified by univariate and multivariate logistic regression analysis. Five common factors were considered to be strongly associated with total and cancer-specific early death, including histological type, tumor size, liver metastasis, surgery, and chemotherapy. Differently, race and brain metastasis were independent risk factors for cancer-specific early death. Tables 2, 3 demonstrate the result of univariate and multivariate logistic regression analysis.

**Table 2:** The univariable and multivariate logistic regression analysis of total early death in the training cohort.

Variable	Univariate analysis			Multivariate analysis		
	OR	95% CI	*P* value	OR	95% CI	*P* value
**Age (years)**						
<67	Ref			Ref		
67–80	1.665	1.208–2.293	0.002	1.487	0.989–2.236	0.056
>80	3.472	2.379–5.067	<0.001	1.624	0.998–2.643	0.051
**Sex**						
Male	Ref			Ref		
Female	1.393	1.030–1.883	0.031	1.128	0.758–1.678	0.552
**Race**						
White	Ref			–		
Black	0.728	0.455–1.165	0.185	–	–	–
Other	0.684	0.360–1.300	0.246	–	–	–
**Histologic type**						
TCC	Ref			Ref		
PTCC	0.560	0.411–0.761	<0.001	0.497	0.333–0.740	0.001
**Size**						
<45 mm	Ref			Ref		
45–73 mm	1.398	0.895–2.183	0.141	1.313	0.750–2.297	0.341
>73 mm	2.145	1.284–3.586	0.004	2.776	1.432–5.383	0.003
Unknown	1.841	1.234–2.746	0.003	1.500	0.903–2.493	0.118
**Grade**						
GI/II	Ref			–		
GIII	0.913	0.396–2.106	0.832	–	–	–
GIV	0.694	0.310–1.554	0.375	–	–	–
**T stage**						
T1	Ref			Ref		
T2	0.716	0.498–1.030	0.072	1.090	0.679–1.751	0.721
T3	0.522	0.307–0.887	0.016	0.945	0.448–1.993	0.881
T4	0.799	0.516–1.237	0.313	1.385	0.769–2.493	0.278
**N stage**						
N0	Ref			–		
N1	0.691	0.439–1.087	0.110	–	–	–
N2	0.830	0.581–1.183	0.302	–	–	–
N3	0.644	0.384–1.081	0.096	–	–	–
**Bone metastasis**						
No	Ref			–		
Yes	1.267	0.954–1.683	0.102	–	–	–
**Lung metastasis**						
No	Ref			Ref		
Yes	1.384	1.043–1.837	0.024	1.396	0.968–2.015	0.078
**Liver metastasis**						
No	Ref			Ref		
Yes	3.058	2.162–4.352	<0.001	3.205	2.052–5.007	<0.001
**Brain metastasis**						
No	Ref			–		
Yes	1.393	0.661–2.934	0.383	–	–	–
**Surgery**						
No	Ref			Ref		
TURBT	0.344	0.220–0.536	<0.001	0.416	0.233–0.742	0.003
RC	0.074	0.034–0.161	<0.001	0.077	0.030–0.199	<0.001
**Radiotherapy**						
No	Ref			–		
Yes	0.914	0.658–1.270	0.594	–	–	–
**Chemotherapy**						
No	Ref			Ref		
Yes	0.079	0.057–0.111	<0.001	0.074	0.050–0.108	<0.001

**Table 3:** The univariable and multivariate logistic regression analysis of cancer-specific early death in the training cohort.

Variable	Univariate analysis			Multivariate analysis		
	OR	95% CI	*P* value	OR	95% CI	*P* value
**Age (years)**						
<67	Ref			Ref		
67–80	1.349	0.972–1.872	0.073	1.274	0.830–1.957	0.268
>80	2.820	1.926–4.128	<0.001	1.487	0.909–2.434	0.114
**Sex**						
Male	Ref			–		
Female	1.324	0.976–1.797	0.072	–	–	–
**Race**						
White	Ref			Ref		
Black	0.729	0.446–1.190	0.206	0.480	0.252–0.911	0.025
Other	0.328	0.135–0.794	0.014	0.194	0.066–0.569	0.003
**Histologic type**						
TCC	Ref			Ref		
PTCC	0.688	0.507–0.934	0.017	0.565	0.376–0.849	0.006
**Size**						
<45 mm	Ref			Ref		
45–73 mm	1.480	0.921–2.378	0.105	1.378	0.757–2.508	0.294
>73 mm	1.995	1.149–3.465	0.014	2.552	1.252–5.202	0.010
Unknown	2.069	1.345–3.184	0.001	1.937	1.117–3.360	0.019
**Grade**						
GI/II	Ref			–		
GIII	0.834	0.357–1.952	0.676	–	–	–
GIV	0.562	0.247–1.276	0.168	–	–	–
**T stage**						
T1	Ref			Ref		
T2	0..662	0.458–0.956	0.028	0.876	0.540–1.420	0.590
T3	0.516	0.301–0.886	0.016	1.240	0.578–2.660	0.581
T4	0.725	0.465–1.130	0.156	1.184	0.651–2.150	0.580
**N stage**						
N0	Ref			–		
N1	0.754	0.470–1.211	0.243	–	–	–
N2	0.789	0.547–1.139	0.207	–	–	–
N3	0.595	0.346–1.021	0.060	–	–	–
**Bone metastasis**						
No	Ref			–		
Yes	1.233	0.920–1.651	0.161	–	–	–
**Lung metastasis**						
No	Ref			–		
Yes	1.215	0.907–1.628	0.192	–	–	–
**Liver metastasis**						
No	Ref			Ref		
Yes	2.973	2.097–4.217	<0.001	3.464	2.188–5.483	<0.001
**Brain metastasis**						
No	Ref			Ref		
Yes	2.226	1.045–4.743	0.038	2.835	1.067–7.537	0.037
**Surgery**						
No	Ref			Ref		
TURBT	0.395	0.252–0.621	<0.001	0.519	0.284–0.948	0.033
RC	0.083	0.038–0.182	<0.001	0.081	0.030–0.220	<0.001
**Radiotherapy**						
No	Ref			–		
Yes	0.876	0.622–1.233	0.447	–	–	–
**Chemotherapy**						
No	Ref			Ref		
Yes	0.073	0.051–0.103	<0.001	0.059	0.039–0.089	<0.001

### Nomogram construction

**Figure 3:**
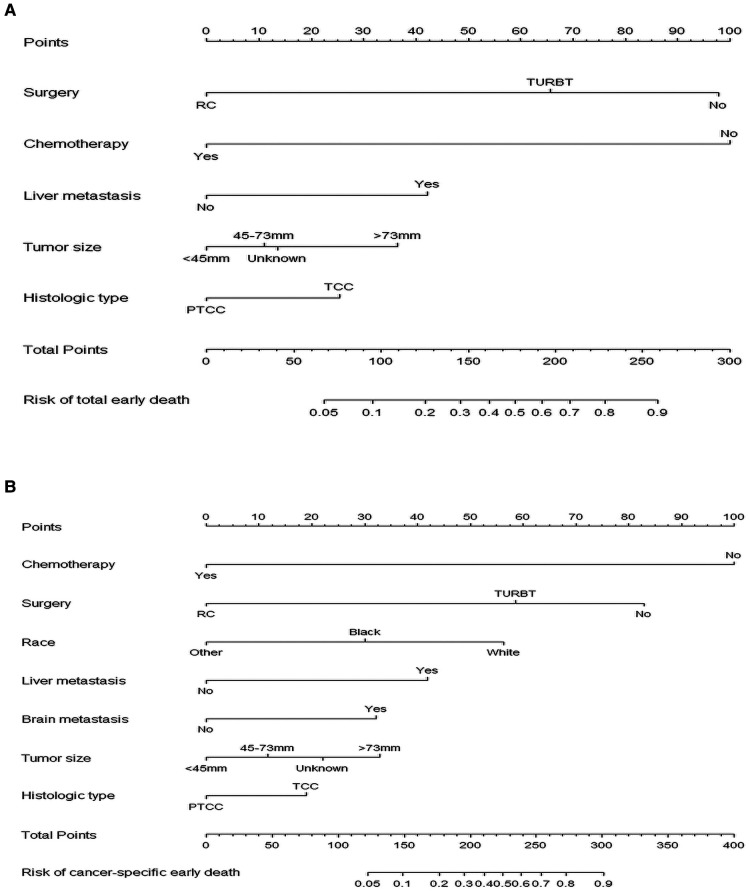
The nomograms of early death in patients with metastatic bladder cancer. (**A**) The total early death; (**B**) The cancer-specific early death.

After identifying relevant variables by univariate regression analysis and multivariate regression analysis, nomograms for total early death and cancer-specific early death were constructed. The incidence of early death in MBC patients can be predicted by calculating a sum of the scores for each risk factor (Figure 3).

### Nomogram validation

**Figure 4:**
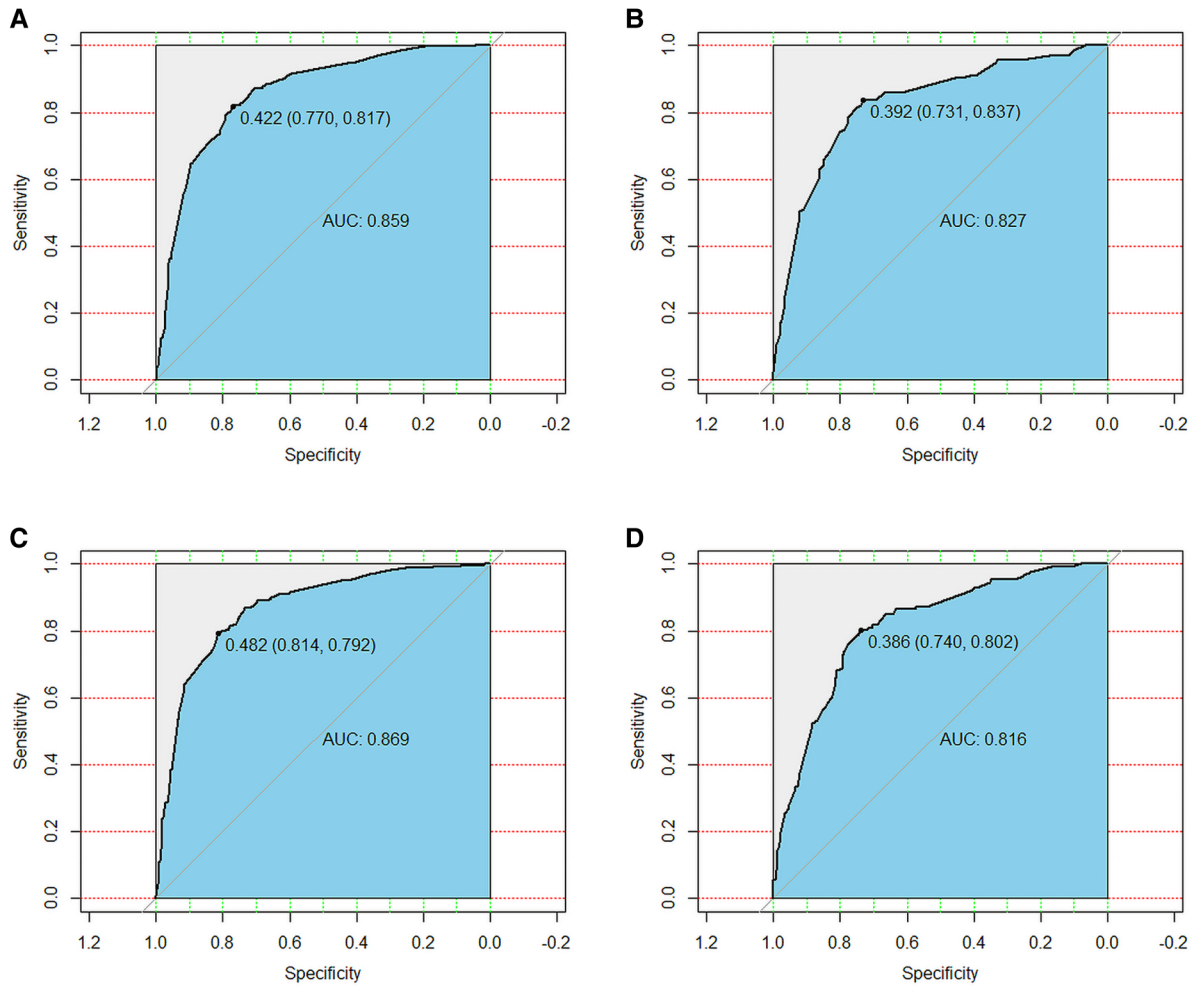
The receiver operating characteristic (ROC) curve for nomogram. (**A**) The training cohort of total early death; (**B**) The validation cohort of total early death; (**C**) The training cohort of cancer-specific early death; (**D**) The validation cohort of cancer-specific early death. AUC, area under the curve; ROC, receiver operating characteristic.

**Figure 5:**
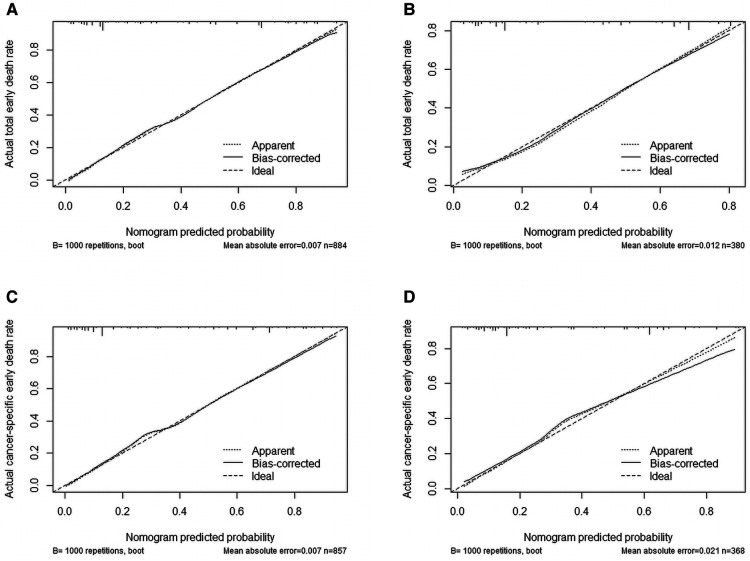
Internal verification plots of nomogram calibration curves by bootstrapping with 1,000 resamples. (**A**) The training cohort of total early death; (**B**) The validation cohort of total early death; (**C**) The training cohort of cancer-specific early death; (**D**) The validation cohort of cancer-specific early death.

**Figure 6:**
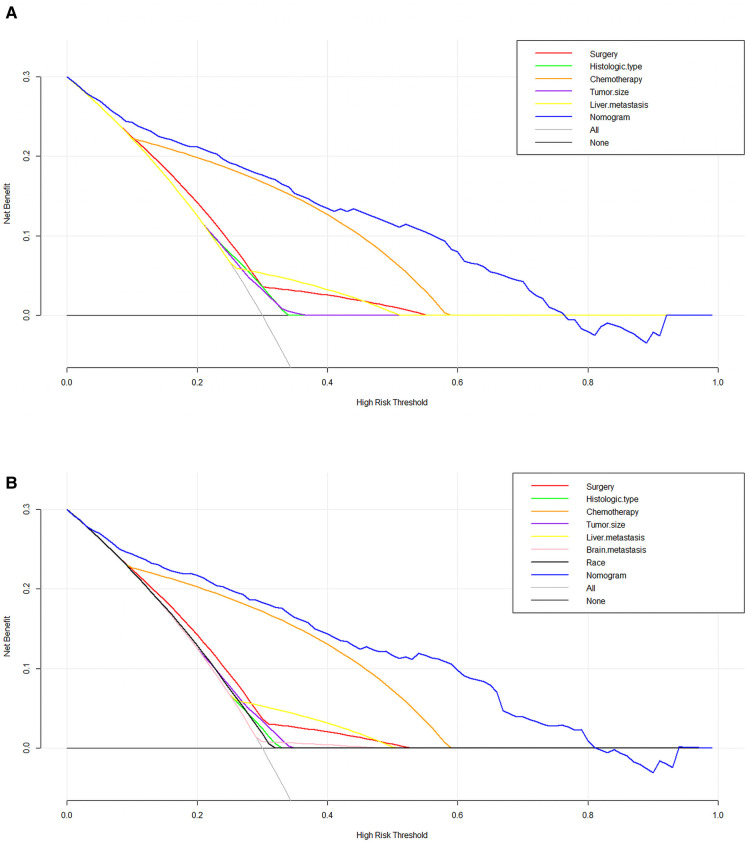
The decision curve analysis (DCA) curve for nomogram. (**A**) The total early death; (**B**) The cancer-specific early death.

**Figure 7:**
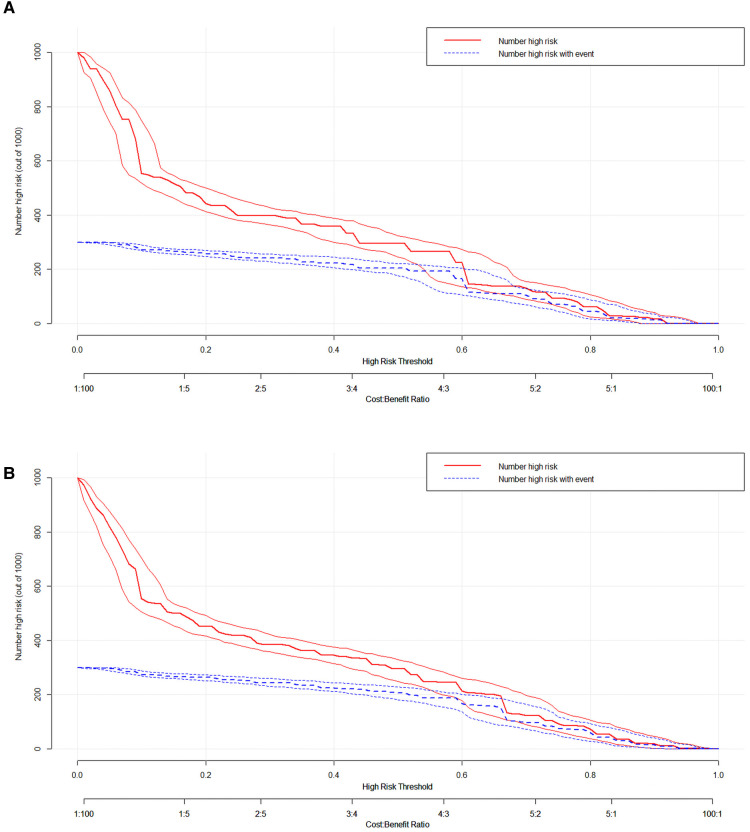
The clinical impact curve (CIC) curve for nomogram. (**A**) The total early death; (**B**) The cancer-specific early death.

We used ROC curve, calibration plot, DCA and CIC to validate the nomograms. According to the ROC curves, the AUC value for total premature death in the training cohort was 0.859 and for cancer-specific premature death was 0.869; the AUC value for total premature death in the validation cohort was 0.827 and for cancer-specific premature death was 0.816 Figure 4). In the calibration curves for both total premature death and cancer-specific premature death, the solid lines were all close to 45° for both the training and validation cohorts (Figure 5), showing the reliability of the models. Finally, compared with other factors, the DCA and CIC of both the training and validation cohorts yielded excellent net clinical benefit (Figures 6, 7).

## Discussion

Bladder cancer is a highly heterogeneous disease, which shows a difference in prognosis. The prognosis of non-muscle invasive bladder cancer is generally favorable, with a 5-year recurrence-free survival rate of 23% and a 5-year progression-free survival rate of 54% (11). In contrast, the prognosis is poorer for muscle-invasive bladder cancer, with an overall 5-year survival rate about 36%–48% (12). Due to patients' adherence and treatment limitations, surgical treatment of patients with muscle-invasive bladder cancer is often not as effective as it could be. As previous studies have shown that more than half of patients with muscle-invasive bladder cancer do not receive the radical treatment (13). Even in patients who have undergone radical treatment, 50% of patients will progress to metastatic bladder cancer (14, 15), In addition, previous predictive models have indicated that patients with non-muscle invasive bladder cancer with lymphovascular infiltration (16) and micropapillary tissue (17) are also susceptible to progression to metastatic disease, bringing a poor prognosis. Patients with MBC have a poor prognosis and are often prone to early death. Therefore, to predict early death in MBC patients is a crucial issue that need to be addressed. As far as we know, few people have explored the risk factors of early death in metastatic bladder cancer. In 2014, Aziz et al. established a nomogram predicting 90-day mortality in patients with bladder cancer after radical cystectomy. Their model included age, American Society of Anesthesiologists score, hospital volume, clinically lymphatic metastases and clinically distant metastases (18). Unlike their research, the target population of our study was metastatic bladder cancer patients, which were more prone to early death. The early mortality of metastatic bladder cancer was higher (37% vs. 9.0%). In addition, our research was based on a larger sample size (1,264 vs. 597). The prediction accuracy of our model was higher (0.859 vs. 0.788). Ultimately, our study showed that larger tumor size, transitional cell carcinoma, liver metastases, lung metastases, and patients who did not undergo surgery and chemotherapy had an increased probability of early death.

According to the latest European Association of Urology guidelines, cisplatin-based chemotherapy is till the first choice for appropriate patients with metastatic bladder cancer. For patients who do not meet the requirements of cisplatin, immunotherapy is recommended for patients with positive programmed death ligand 1 (PD-L1), or carboplatin therapy is recommended for patients with negative PD-L1 (19). Though the long-term results have not been so satisfactory (20), it has been demonstrated in many phase II/III clinical trials that chemotherapy regimens can provide a considerable benefit to patients. In our study, 80.7% of patients did not experience chemotherapy, and in the final nomograms, patients without chemotherapy received the highest risk scores. Our results demonstrated there were still many patients with MBC who neglect the importance of chemotherapy. In the future, clinicians need to strengthen the management of MBC patients with adjuvant chemotherapy. A systematic search of English language literature displayed that the surgical resection of metastatic tumors is technically feasible and can be carried out safely. It may help improve cancer control and ultimately improve the survival rate of specific patients with limited metastatic burden (21). Similar to this, we also found that surgery can improve the prognosis of patients with metastatic bladder cancer to a certain extent, but clinicians still need to strictly set the indications for surgery. In an analysis of metastatic patterns in MBC patients, Shou J, et al. found that patients with bone metastasis accounted for the majority, liver metastasis had the worst prognosis, and it has been demonstrated that there is no significant difference in the overall survival of patients with multiple metastatic sites compared to single metastatic site (22). In our study, bone metastasis and liver metastasis were significant risk factors of early death in MBC patients, which shows good agreement with the above findings.

In recent years, several studies have reported that bladder cancer is associated with the expression of certain genes and proteins, which can be used to determine metastasis, prognosis and treatment of bladder cancer (23), Currently, Smith, et al. has already established a 20 genetic models for identifying bladder cancer patients with high risk of lymph node metastasis (24), in addition, Karyopherin-a2 (KPNA2) interaction with Chromobox 8 (CBX8) has been suggested as an indicator that patients are at high risk of metastasis (25). Due to the SEER database lack the information of molecular markers, our study did not establish a model containing molecular markers. However, it is difficult to popularize the clinical application of molecular markers.

Our nomograms were built on the SEER database and had a large sample size, which ensures the reliability of our results. Through internal validation, our models perform perfect in terms of predictiveness and clinical benefits. As a practical tool, DCA and CIC can be used to check the validity of model-based clinical decisions (26), Our study showed that the net benefit of our nomograms was better than the other two conditions (all screening or no screening). The scores obtained from the analysis of the nomogram can be used to derive a patient's probability of early death, which can be used by clinicians for end-of-life care and by oncologists to design clinical trials. In addition, identifying patients at risk of early death is essential to reduce the burden on patients, because the side effects of treatment and the inconvenience of going to the hospital may outweigh the benefits of treatment.

However, there are some unavoidable limitations of this study. Firstly, a number of known correlated factors were not considered in the nomograms. For example, Karnofsky performance status, functional comorbidities, the aforementioned biomarkers, and some emerging biomarkers such as TNFRSF14AS1, AL354919.2, OCIAD1-AS1 (32) were thought to be associated with MBC prognosis, but SEER database does not include these data. Secondly, our study was developed retrospectively and potential selection bias could have adversely affected the conclusions. Thirdly, although our models were validated internally, our models have not been validated with external clinical data. Future researches could validate it with clinical data to assess its plausibility.

## Conclusion

In summary, two comprehensive nomograms established in our study can accurately predict early death from MBC, which enable surgeons to develop targeted treatment, follow-up strategies and improve survival outcomes for patients with MBC.

## Data Availability

The original contributions presented in the study are included in the article/Supplementary Material, further inquiries can be directed to the corresponding author/s.
